# Supplementary material for: HIVprotI: an integrated web based platform for prediction and design of HIV proteins inhibitors
Source: J Cheminform. 2018 Mar 9;10:12. doi: 10.1186/s13321-018-0266-y (PMC5845081; doi:10.1186/s13321-018-0266-y)
Supplement: Supplementary file 2 — Additional file 2. Source code of HIVProtI web server. [file 13321_2018_266_MOESM2_ESM.zip › HIVprotI_Source-code/hivprotI/weka-3-6-6/doc/weka/classifiers/rules/DTNB.html]

DTNB


---


|  |  |  |
| --- | --- | --- |
| **Overview** \| **Package** \| **Class** \| **Tree** \| **Deprecated** \| **Index** \| **Help** \| **Weka's home** | |  |
| **PREV CLASS**   **NEXT CLASS** | **FRAMES**    **NO FRAMES**     **All Classes** |  |
| SUMMARY: NESTED \| FIELD \| CONSTR \| METHOD | DETAIL: FIELD \| CONSTR \| METHOD |  |


---


## weka.classifiers.rules Class DTNB

```
java.lang.Object
  weka.classifiers.Classifier
      weka.classifiers.rules.DecisionTable
          weka.classifiers.rules.DTNB
```

**All Implemented Interfaces:**: java.io.Serializable, java.lang.Cloneable, AdditionalMeasureProducer, CapabilitiesHandler, OptionHandler, RevisionHandler, TechnicalInformationHandler, WeightedInstancesHandler

---

``` public class DTNB extends DecisionTable ```

Class for building and using a decision table/naive bayes hybrid classifier. At each point in the search, the algorithm evaluates the merit of dividing the attributes into two disjoint subsets: one for the decision table, the other for naive Bayes. A forward selection search is used, where at each step, selected attributes are modeled by naive Bayes and the remainder by the decision table, and all attributes are modelled by the decision table initially. At each step, the algorithm also considers dropping an attribute entirely from the model.
